# A Molecular Evolution Approach to Study the Roles of Tropomyosin in Fission Yeast

**DOI:** 10.1371/journal.pone.0076726

**Published:** 2013-10-22

**Authors:** Susanne Cranz-Mileva, Melissa C. Pamula, Bipasha Barua, Brinda Desai, Yaejee Hannah Hong, Jacquelyn Russell, Richard Trent, Jianqiu Wang, Nancy C. Walworth, Sarah E. Hitchcock-DeGregori

**Affiliations:** 1 Department of Pathology and Laboratory Medicine, Robert Wood Johnson Medical School, Rutgers University, Piscataway, New Jersey, United States of America; 2 Department of Pharmacology, Robert Wood Johnson Medical School, Rutgers University, Piscataway, New Jersey, United States of America; Fondazione Edmund Mach, Research and Innovation Centre, Italy

## Abstract

Tropomyosin, a coiled-coil protein that binds along the length of the actin filament, is a universal regulator of the actin cytoskeleton. We have taken a bioinformatics/proteomic approach to studying structure-function relationships in this protein. The presence of a single, essential tropomyosin gene, *cdc8*, in fission yeast, *Schizosaccharomyces pombe*, enables a systems-based approach to define the residues that are important for cellular functions. Using molecular evolution methodologies we identified the most conserved residues and related them to the coiled coil structure. Mutants in which one or more of 21 of the most conserved surface residues was mutated to Ala were tested for the ability to rescue growth of a temperature-sensitive *cdc8* mutant when overexpressed at the restrictive temperature. Based on altered morphology of the septum and actin cytoskeleton, we selected three sets of mutations for construction of mutant *cdc8* strains using marker reconstitution mutagenesis and analysis of recombinant protein *in vitro*: D16A.K30A, V114S.E117A.H118A and R121A.D131A.E138A. The mutations have sequence-specific effects on cellular morphology including cell length, organization of cytoskeletal structures (actin patches, actin cables and contractile rings), and *in vitro* actin affinity, lending credence to the proteomic approach introduced here. We propose that bioinformatics is a valid analysis tool for defining structure-function relationships in conserved proteins in this model organism.

## Introduction

The actin cytoskeleton has ancient origins. Eukaryotic actin and prokaryotic homologues share an overall folding pattern, a bound nucleotide, and some common protofilament interactions despite poor sequence homology [1], [2]. Within eukaryotes actin shows remarkable sequence and structural conservation; human β-actin and *S. pombe* actin (Act1p) are 90% identical. Just as the actin sequence is conserved, so are the functions and states of the actin cytoskeleton throughout eukaryotes [3]: actin monomers, filaments and bundles that function in cellular motility, intracellular transport, cell shape and formation of the contractile ring during cytokinesis. Accordingly, numerous actin binding proteins and their functions are universal to eukaryotes: myosins, Arp2/3 complex, ADF-cofilin, formins and crosslinking proteins such as α-actinin and fimbrin, to name a few.

One class of actin binding proteins, the tropomyosins (Tm), has been reported only in animals and fungi. Tropomyosin is a two-chained α-helical coiled coil that binds end-to-end along the helical actin filament, stabilizing it and regulating its interactions with other proteins [4], [5]. Tropomyosin regulates diverse actin filament functions including myosin-dependent contraction and motility in muscle, yeast and animal non-muscle cells. By binding and stabilizing actin filaments and protecting them from severing, branching by Arp2/3 complex and from certain crosslinkers, Tm controls actin filament dynamics. It can positively regulate formins [6], [7], [8], [9] and make the interaction of myosin with actin cooperative and in some cases processive [10], [11], [12], [13].

We postulate that residues required for universal Tm functions are conserved during evolution. In order to test this hypothesis we have taken a bioinformatics/proteomic approach to studying structure-function relationships in this protein using molecular evolution methodologies to construct phylogenetic trees. Based on the trees, the substitution rates at individual codons are calculated to identify the most conserved sites. In a previous study we constructed phylogenetic trees for coding regions of animal Tm genes and showed that the most conserved residues in striated muscle Tm have roles in actin binding and actomyosin regulation, two Tm functions that can be measured *in vitro* [14], [15], [16]. The results have been used to test and modify available thin filament models [14], [16], [17], [18], [19]. Other cellular Tm functions, such as assembly of higher order structures, regulation of cellular protrusions, vesicular transport, cellular polarity determination and regulation of cellular contraction and cytokinesis are not readily analyzed *in vitro*. The combination of bioinformatics and proteomics to analyze function in a model system offers such an opportunity.

The complexity of the mammalian Tm gene family that results from four genes, alternate promoters and alternatively expressed exons encoding 40 or more isoforms [20] makes *in vivo* or even *in situ* studies impractical. For this reason, we turned to the fission yeast model organism, *Schizosaccharomyces pomb*e that has a single, essential Tm gene, *cdc8* [21], [22]. Most classes of actin binding and regulatory proteins are represented in fission yeast, but the number of isoforms is small compared to vertebrates (1 actin, 5 myosins, and 2 formins, for example). Nevertheless, they account for the formation of the three actin structures in vegetative cells: cables, contractile rings and patches [23]. Fission yeast Tm (Cdc8p) is predominantly localized in actin cables and contractile rings with smaller amounts in actin patches [21], [24], [25]. Although there is only one Tm isoform, post-translational N-terminal acetylation affects actin affinity *in vitro* and localization within the cell [25], [26], adding a mechanism for diversity beyond the genomic simplicity.

Here we present the first stage in the implementation of a proteomic approach to investigate structure-function relationships in Tm regulation of the actin cytoskeleton in fission yeast, using the organism as a bioassay. We identified the most evolutionarily conserved residues and made mutations in the 21 most conserved surface residues that are most likely to be involved in binding other proteins. We screened for cellular function by assaying the ability of each mutant protein, when overexpressed, to rescue growth of a *cdc8^ts^* mutant at the restrictive temperature. Although all rescued growth, certain mutants had morphological defects that infer altered assembly of cytoskeletal structures as well as determination of cellular polarity. Based on the initial screen, we selected three mutants for further study with the creation of mutant *cdc8* strains using homologous gene replacement and *in vitro* analyses of recombinant protein. The mutations affect the cellular morphology in specific ways including cell length, organization and positioning of actin cytoskeletal structures and change the *in vitro* actin affinity. The results establish the validity of the proteomic approach for the study of structure-function relationships and set a framework to establish the molecular basis of actin regulation by tropomyosin in a cellular system.

## Results

The first step in our bioinformatics approach to the study of structure-function relationships in fission yeast Tm, Cdc8p, was to identify the most conserved residues using molecular evolution methodology. We then tested the importance of conserved surface residues for cellular function using overexpression and gene replacement strategies, as well as *in vitro* functional assays. The report here establishes the validity of this proteomics approach as a way to screen for functionally important residues and paves the way to a detailed dissection and understanding of cytoskeletal mechanisms in fission yeast.

### Evolutionary Analysis

In our evolutionary analysis of animal Tm genes we searched for homologous sequences in protein databases in an attempt to determine how deep in the phylogenetic tree we could identify Tm [15]. A BLAST search using the N-terminal 14 residues of rat striated muscle α-Tm, a highly conserved region in animal Tms, identified a sequence in *Phaeosphaeria nodorum* that encodes a 161 amino acid protein, the length of known fungal Tms, with a coiled coil propensity similar to confirmed Tms. Searching further with residues 1–24 of *Phaeosphaeria nodorum* revealed other known fungal Tm sequences. After aligning the sequences that were annotated as Tms, we selected conserved regions of 40 amino acids that we used to search the NCBI database using BLAST for additional fungal Tm sequences. Using this search method, we collected and evaluated 29 expressed sequences from 27 fungal species that had annotated genomic data available (as of the end of 2008, Table S1in File S1). Requirements for inclusion were conservative and included location of the known coding sequence at the 5′ end of the open reading frame, a length consistent with known Tm sequences, and the absence of internal proline. We were unable to identify homologous sequences in protists or plants. The redundancy of the coiled coil sequence heptapeptide repeat limited the depth of the search.

**Figure 1:**
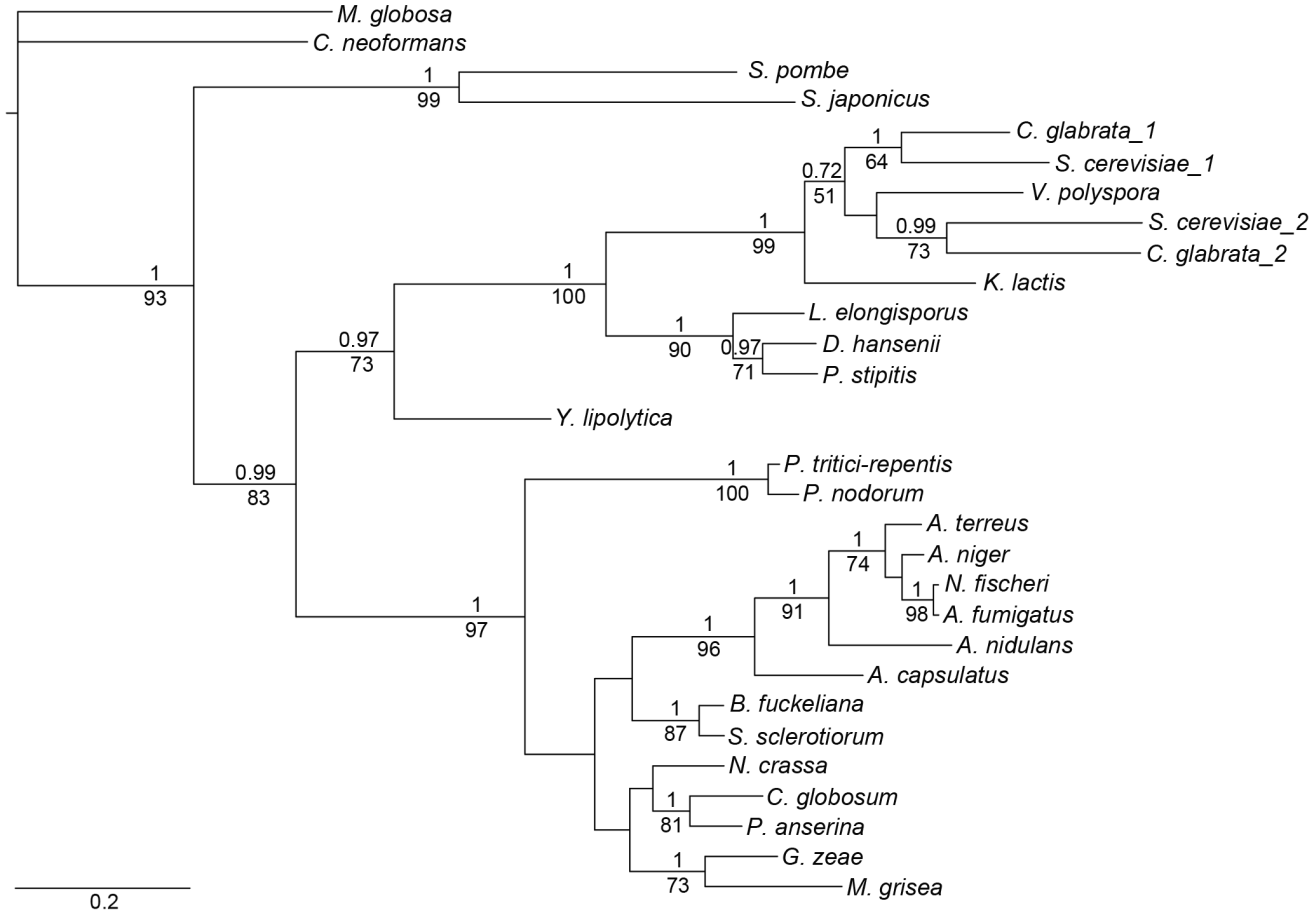
Fungal tropomyosin phylogenetic tree based on Bayesian analysis. The topology of the maximum-likelihood tree showed comparable results. Bayesian posterior probabilities and maximum-likelihood bootstrap values are measures of the probability that the observed branches are “true”, and are shown above and below the branches, respectively. All other branches had >50% posterior probabilities but were not as well supported in the maximum-likelihood analysis (bootstrap values<50%). Scale bar indicates branch length (substitutions per site).

The protein sequences were aligned for phylogenetic tree construction (Figure S1 in File S1). All species have a gene encoding a 161-residue protein, a length sufficient to span four subunits in the actin filament. Two species, *N. glabrata* and *S. cerevisiae* have two genes that encode proteins of 161 and 199 residues, respectively. The longer form reflects an insertion of 38 residues after codon 35 and corresponds to the addition of an actin binding period, enabling the longer form to span five actin subunits in the filament. The assembled dataset was used to construct trees using maximum-likelihood (GARLI 1.0) [27] and Bayesian (MrBayes v3.2) [28] approaches for phylogenetic analysis (Figure 1, Bayesian tree). The consensus trees from the two approaches resulted in similar topologies with good branch support, >60% posterior probability for all major branches (except one 52% node) and bootstrap values >60% (except one 51% node). The tree corresponds well with known fungal phylogeny (http://tolweb.org/Fungi/).

**Figure 2:**
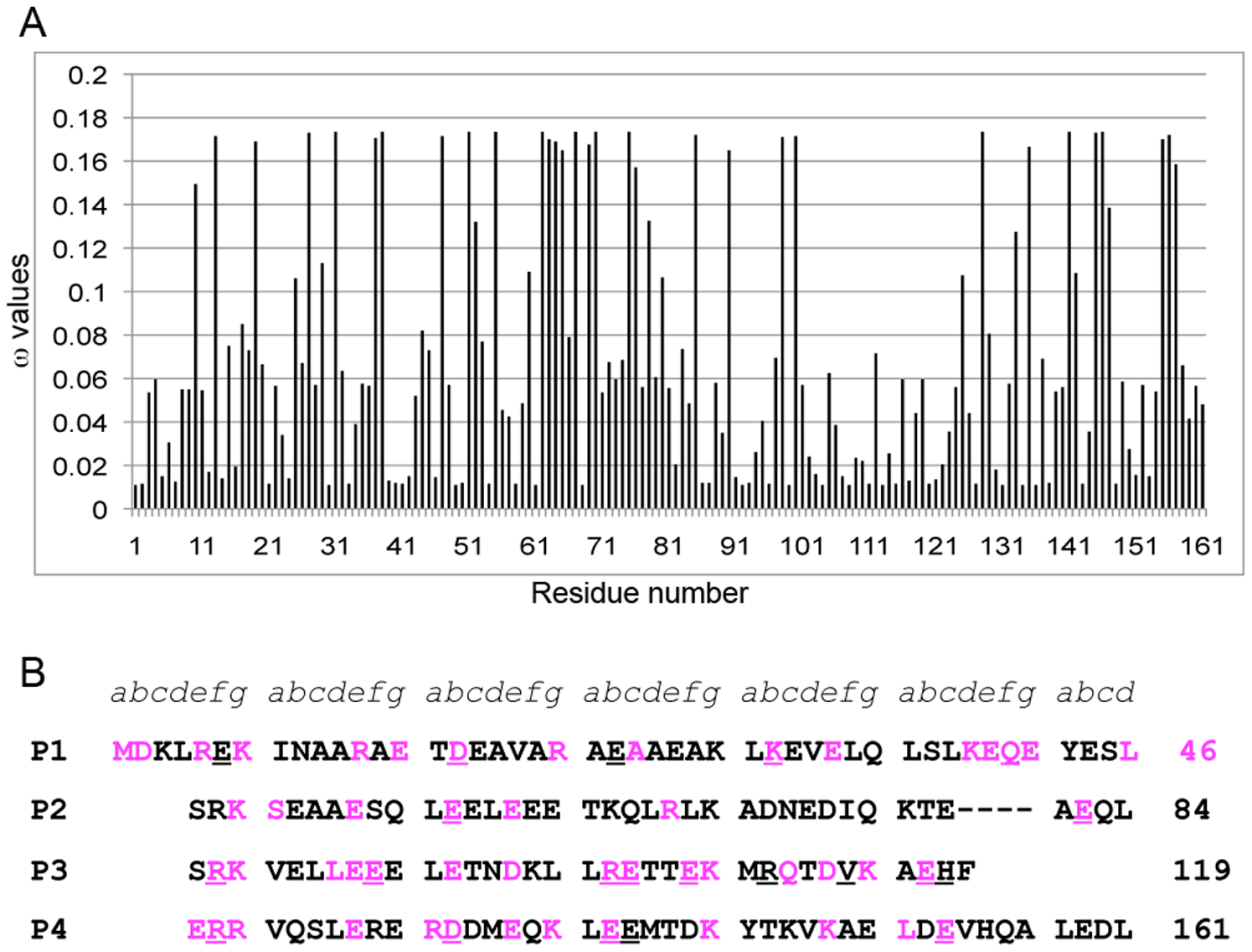
Evolutionary analysis of *S. pombe* tropomyosin (Cdc8p). A. Average ω values of the 161 codons of the Cdc8p sequence, where ω = dN/dS, and dN, dS are the nonsynonymous (encodes a different amino acid) and synonymous (encodes the same amino acid) substitution rates for codons, respectively. ω<1 represents negative selection, ω = 1, neutral selection, and ω>1, positive selection. The ω values for all codons of Cdc8p were<1 indicating negative selection and a highly conserved protein. The codons with average ω≤0.02 (an arbitrary value) were selected as conserved sites. B. The Cdc8p sequence showing the conserved residues (average ω≤0.02) in magenta. *a–g* are the positions in the heptad repeat and - - - - is the interruption in the heptad repeat (stammer) found in fungal tropomyosins. The sequence is displayed in the length of periods (P1–P4) proposed for vertebrate tropomyosins (Phillips, 1986). The positions of the 21 mutated sites are underlined.

Based on the trees, the rates of evolution at individual codons were determined using PAML 4.1 [29] where ω = dN/dS, dN and dS are the nonsynonymous and synonymous substitution rates for codons, respectively. ω<1 represents residues under negative selection, ω = 1, neutral selection, and ω>1, positive selection. We plotted the ω values against the 161 codons of *cdc8* (Figure 2A; Table S2 in File S1. Tropomyosin is highly conserved overall since ω<0.2 for all residues. The codons with average ω≤0.02 (an arbitrary cutoff, ∼10% of the maximal ω) were selected as highly conserved sites and are highlighted in the *S. pombe cdc8* protein sequence (Figure 2B).

The distribution of the highly conserved residues was similar in the fungal and animal bioinformatics analyses [15]. Of the sites, 31.2% (50 residues of 160, exclusive of the initial methionine) were highly conserved in fungi, 31.8% in animals. Considered in terms of the heptapeptide “*a,b,c,d,e,f,g*” repeat characteristic of coiled coils, the largest fraction was in *e* and *g* residues, often paired oppositely charged residues involved in inter-chain ion pairs in the coiled coil (15.6% in fungi, 12.4% in animals). The *a* and *d* residues that form the hydrophobic core between the two chains of the coiled coil are 3.8% evolutionarily-conserved in fungi and 8.5% in animals. The difference reflects the lower thermal stability of Cdc8p relative to mammalian striated muscle Tms (T_M_ = 33.1°C for AS-Cdc8p vs 49.4°C for AS-rat skeletal α-Tm; [30], commensurate with a lower growing temperature. The core may be more critical for conserving structure and function in animal than in fungal Tms: the animal Tms have conserved interface Ala clusters and a conserved charged residue at a *d* position (D137) that is not present in fungal Tms. Fungal Tms have a four-residue interruption (a stammer) in the heptad repeat [31] that would alter interface packing and the supercoil [32]. On the other hand, the larger fraction of conserved *e* and *g* sites in fungi may partially compensate by contributing to the stability of the coiled coil. Both animal and fungal Tms have similar fractions of conserved *b, c,* and *f* surface sites (11.9% in fungi, 11.0% in animals), residues that are most available for interacting with other proteins and are therefore the focus of our proteomic screens [14], [15], [16], and the present work. Despite the above parallels with animal Tms, the fungal and animal Tms are too evolutionarily distant for meaningful alignment.

### Proteomic Screen: Rescue of a *cdc8* ^ts^ Mutant at Restrictive Temperature

**Figure 3:**
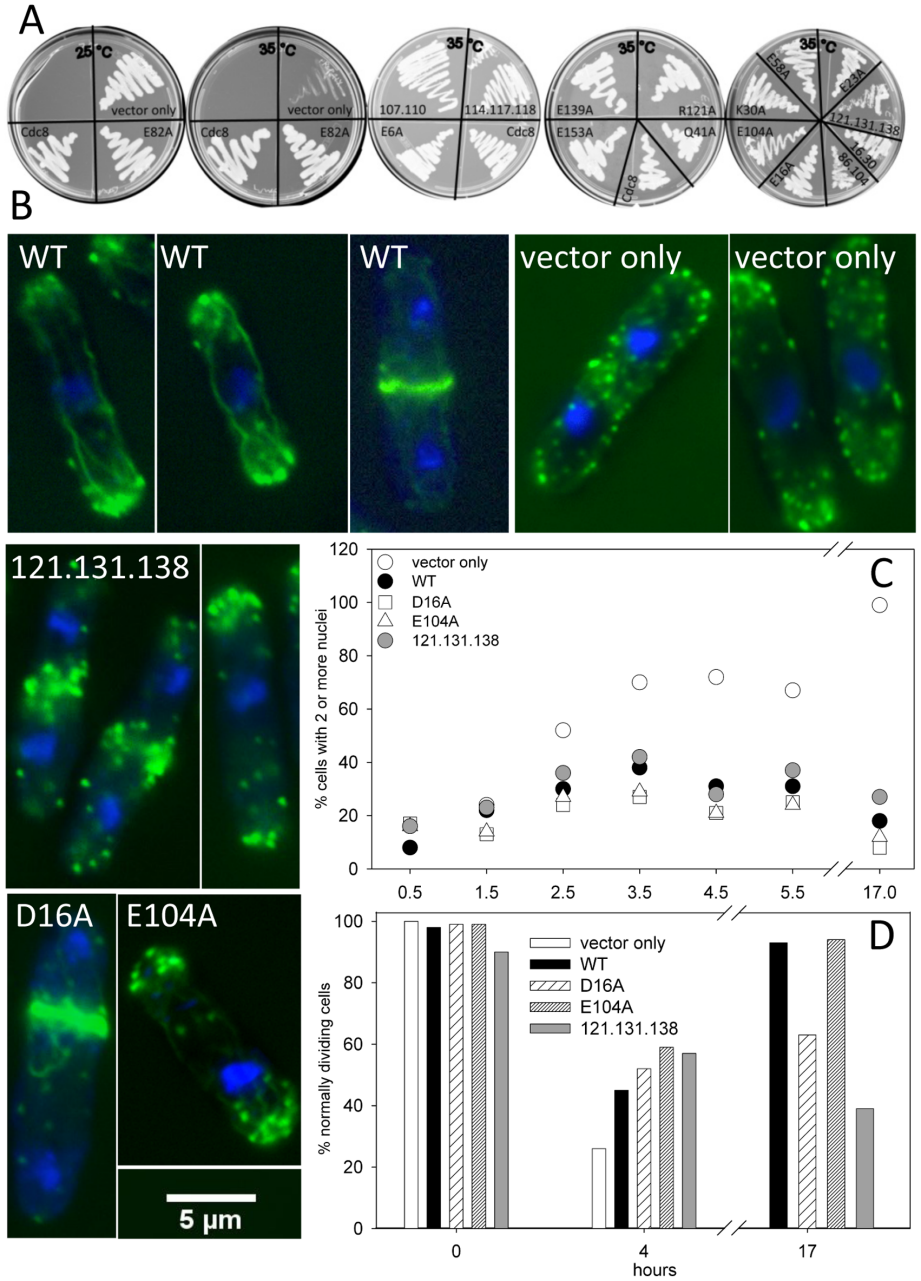
Proteomic screen: Rescue of *cdc8-27* by pREP41X-*cdc8*. A. *Cdc8-27* cells were transfected with pREP41X (vector alone) or expressing wildtype or mutant Cdc8p and grown on EMM agar, lacking thiamine. The plate on the left was grown at 25°C, the four others were grown at the restrictive temperature, 35°C. B, C, D. Analysis of cells over-expressing wildtype Cdc8p and three Cdc8p mutants: D16A, E104A, and R121A.D131A, E138A in *cdc8-27*. Cells were grown to mid-log phase in EMM medium with thiamine, and transferred to EMM medium without thiamine and grown overnight at 25°C. Cells from the overnight culture were transferred to the restrictive temperature (35°C). Samples were taken at times after transfer and fixed. Cultures were maintained in the mid-log phase of growth. B. The cells for Alexa Fluor-488/DAPI- stained images were fixed at 4 hr (vector only, wildtype and E104A) or 27 hours (D16A, R121A.D131A.E138A) after transfer to 35°C. The images are of cells selected to show the types of observed abnormalities. C, D. The cells were fixed and stained with Calcofluor and DAPI and analyzed for the number of nuclei and the appearance of the septum in cells with two nuclei C. Percent of cells with ≥2 nuclei. At least 200 cells were counted for each time point. D. Percent normally dividing cells: Percent of binuclear septated cells showing a single, medial well-defined septum that extends from one side to the other. At least 50 cells were counted for each time point.

Our initial screen for the functional importance of conserved *cdc8* sites was to test the ability of mutant protein to rescue a *cdc8* ^ts^ mutant at the restrictive temperature. Based on the evolutionary analysis (ω≤ 0.02, or close to the value), evaluation of the amino acid sequences for conservation of amino acid class, and our previous results with vertebrate Tms [14], [15], [16], we selected 21 surface sites for mutagenesis, underlined in Figure 2B. All sites are *b, c* or *f* positions in the coiled-coil heptad repeat because they are the most likely to be involved in binding other proteins and the least likely to influence folding and stability. We introduced one or more Ala mutations (except Val114Ser) into *cdc8* cloned in the pREP41x vector under control of the thiamine-repressible *nmt* promotor [33], [34] for overexpression in *S. pombe* to generate a set of 16 mutants (Table S3 in File S1). The mutants are E6A, D16A, E23A, D16A.K30A, K30A, Q41A, E58A, E82A, E86A.E93A.R103A.E104A, E104A, E107A.R110A, V114S.E117A.H118A, R121A, R121A.D131A.E138A, E139A, and E153A). Overexpression of wildtype and mutant Cdc8p rescued growth of the ts mutant, *cdc8-27* (TP9 strain) [35] at the restrictive temperature, though R121A.D131A.138A grew more slowly (35°C, Figure 3A). In another study E6A and D16A rescued growth of *cdc8-110*, another temperature sensitive *cdc8* mutant, at the restrictive temperature, but growth with D16A was somewhat slower than wildtype [30]. The transfection of wildtype and several Cdc8p mutants into wildtype *S. pombe* (SP6 strain) had no obvious effects on growth or morphology, either on agar or in suspension in the absence of thiamine.

Despite the ability to rescue growth, overexpression of mutant protein in the ts strain (*cdc8-27*) resulted in a variety of phenotypes that include abnormal contractile ring and septum formation, actin cable organization and nuclear and septum localization, summarized in Table S3 in File S1. Figure 3C shows the results for the ts mutant (vector only) and for rescue by overexpression of wildtype and three Cdc8p mutants. In all, the percentage of cells with ≥2 nuclei increases during the first 3–4 hours after transfer to the restrictive temperature, the time required for the first cell division. After that, the percentage of cells with ≥2 nuclei continues to increase in cells with vector only because *cdc8-27* cells become multinucleated being unable to undergo cytokinesis, while all others return to normal or near normal values (Figure 3C; Table S3 in File S1). Cells expressing the following Cdc8p mutants have a higher percentage of cells in division: R86A.E93A.R103A.E104A, R121A, and R121A.D131A.E138A, but most dividing cells have two nuclei after 17–24 hours. The percentage of cells with >2 nuclei was variable, complicated by the presence of some cells that we presume lost the vector and reverted to the multinucleated ts phenotype.

To screen for correct formation and localization of the septum, we stained the cells with Calcofluor, together with DAPI to visualize the nuclei. Figure 3D shows the fraction of normally dividing, septated cells with normal septa at 25°C (t = 0), and at times after transfer to the restrictive temperature for overexpression of three of the mutants. At t = 0 nearly all septa were normal. After 4 hours, ∼30% of the cells with ‘vector only’ had normal septa. In cells over-expressing wildtype or mutant Cdc8p, after 4 hours there were ∼50% normal septa reflecting the residual ts phenotype (multiple, poorly organized septa and abnormal morphologies). After 17–24 hours, almost no *cdc8-27* cells were normal; most had >2 nuclei, abnormal and multiple septa, and were branched. By 17 hours most cells expressing wildtype and E104A had normal septa while D16A and R121A.D131A.E138A had an elevated fraction of cells with an abnormal morphology and number of septa (Figure 3D). Certain other mutants divided normally based on nuclear number, yet had a higher percentage of abnormalities including shape, position or number of septa (E6A, D16A, E107A.R110A, for example; Table S3 in File S1). Two mutants (E82A, V114S.E117A.H118A) were so variable from one transformation to the next that we did not report values in Table S3 in File S1. In general, however, overexpression of mutant Cdc8p had poor penetrance; most cells were morphologically similar to wildtype.

Since Cdc8p is required for actin cable and contractile ring formation, we stained the cells with phalloidin to visualize the actin cytoskeleton after transfer to the restrictive temperature (Figure 3B). Cells overexpressing wildtype Cdc8p showed long straight actin cables, polar distribution of actin patches and a discrete contractile ring typical of the *S. pombe* actin cytoskeleton. In *cdc8-27* (with empty vector), after four hours at 35°C, when many cells still have one or two nuclei, there were no contractile rings or actin cables, and the polarization of actin patches was poor, as others have reported with this and other *cdc8^ts^* mutants [36], [37], [38]. Most cells overexpressing Cdc8p mutants had normal actin cytoskeletons, but there was variability, including poorly organized actin cables, failure to assemble a normal contractile ring, abnormal positioning of nuclei and the contractile ring or weak polarization of actin patches, depending on the Cdc8p mutant. One mutant, R121A.D131A.E138A, had a consistent phenotype: poorly formed cables, failure to form a coherent contractile ring and irregular polarization of actin patches. Nevertheless, these cells could complete cell division because cells with >2 nuclei were rare after 17–24 hours at 35°C.

While we were disappointed that overexpression of mutant Cdc8p did not result in more consistent and severe phenotypes, we did find morphologies associated with specific mutations, implying that the sites mutated are involved in specific Cdc8p functions. The penetrance of abnormal phenotypes resulting from overexpression of Cdc8p mutants was generally poor but the results aided in the selection of sites for further study using gene replacement and *in vitro* analysis of recombinant protein. As Tm is a two-chained coiled coil, we expect the mild phenotypes result from heterodimer formation of molecules with one chain encoded by *cdc8-27* and one by the pREP-*cdc8* vector. Immunoblots showed that Cdc8p was present in *cdc8-27* cells after 24 hours at the restrictive temperature and therefore available for heterodimer formation. The only mutant with a consistent phenotype (R121A.D131A.E138A) has sites close to the *cdc8-27* mutation (E129K, confirmed in our laboratory) [22], which may explain the absence of a functional heterodimer. Reasonably functional Cdc8p could be formed with heterodimers of Cdc8-E129K with the other mutant proteins. We suggest that E16A is less effective than wildtype in rescuing growth of *cdc8-110* at the restrictive temperature, as reported by [30], because *cdc8-110* has mutations in the vicinity of Glu16 (A18T, E31K;Hitchcock-DeGregori, unpublished) that may impair formation of a functional heterodimer.

### Proteomic Screen: Gene Replacement to Test Phenotypes of Site-specific *cdc8* Mutants

#### Strategy

The construction of strains carrying mutations in the *cdc8* gene allows us to test the hypothesis that mutation of conserved sites will result in specific phenotypes. Based on our results with rescue of the ts phenotype (Figure 3, Table S3 in File S1), we selected three sets of mutations to introduce into the *cdc8* gene: D16A.K30A, V114S.E117A.H118A, and R121A.D131A.E138A, hereafter referred to as 16.30, 114.117.118 and 121.131.138. Making mutations in the *cdc8* gene overcomes the problem of heterodimer formation with an endogenous Tm as well as the generic caveats with overexpression. Furthermore creation of mutant *cdc8* strains will enable future genetic experiments.

**Figure 4:**
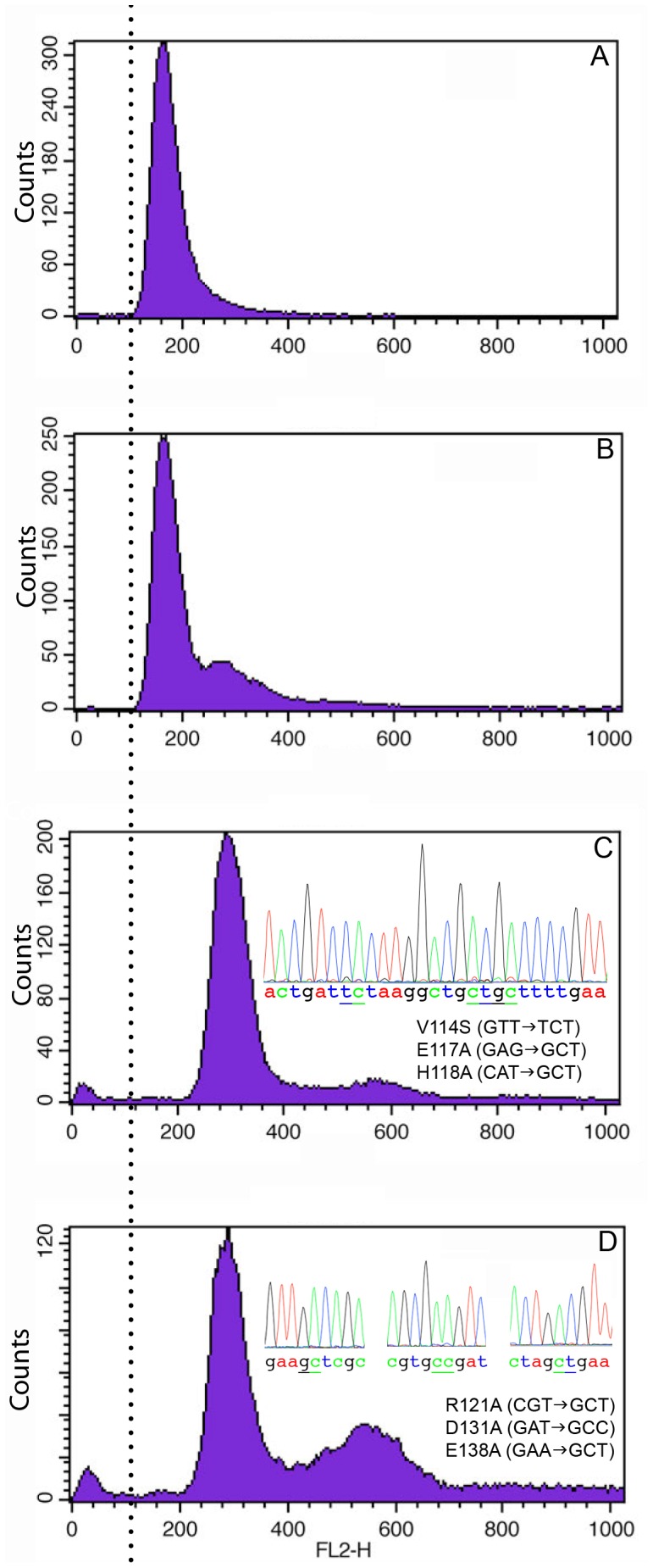
FACS analysis of gene replacement strains. FACS analysis (courtesy of Matthew O’Connell, Mt. Sinai School of Medicine) showed that wildtype and 16.30 strains (A and B) are haploid and 114.117.118 and 121.131.138 strains (C and D) are diploid. With 121.131.138 about 5% of the FACS signal is off scale (signal >1000), and these are mostly clumped cells, based on size. Notice the presence of a second peak indicating a delay in cell division in the three mutant strains. The relative size of the secondary peak is greatest for 121.131.138 (panel D). The FACS analysis of 16.30 was of a colony from the original isolate that was a spontaneous haploid, which arose after the completion of the study. The 16.30 cells in all the morphological studies were diploid. We verified the mutations in the genomic sequences of all strains and determined that the diploid strains are homozygous. All strains were grown to mid-log phase in YEA at 30°C.

Because *cdc8* is an essential gene, we took advantage of the facility of homologous recombination in *S. pombe* and used marker reconstitution mutagenesis, a reverse genetic approach developed to create mutant strains [39]. Whereas the approach has been used to introduce conditionally-defective (ts) alleles into specific genes [39], [40], we are using directed, specific mutagenesis and therefore have no selection method other than the nutritional marker (*his5^+^*). We first constructed a strain that integrated *his5Δc:ura4^+^* next to the 3′ end of the coding region of *cdc8* in a *his5-D1 ura4-D18* strain (Figure S2 in File S1). Using homologous recombination, we then incorporated *cdc8* (wildtype or with the desired mutations) and the C-terminal region of *his5* (*his5^c^*) to place *his5^+^* proximal to *cdc8*. Recombinants were selected for the ability to grow in the absence of histidine and uracil. Mutants were backcrossed twice to parental strains with *cdc8^+^* or *cdc8-27* to ensure tight and stable linkage to *his5^+^* and *cdc8*. The presence of mutations was confirmed by genomic sequencing of the entire *cdc8* gene. FACS analysis indicated that the114.117.118 and 121.131.138 strains are diploid and homozygous (Figure 4). We have been unable to isolate these two mutants as haploid strains. The 16.30 strain isolated and characterized in the analyses described below was diploid. The FACS analysis of 16.30 in Figure 4 was of a colony from the original isolate, which was a spontaneous haploid that arose after completion of the rest of the study.

#### Cell morphology

**Figure 5:**
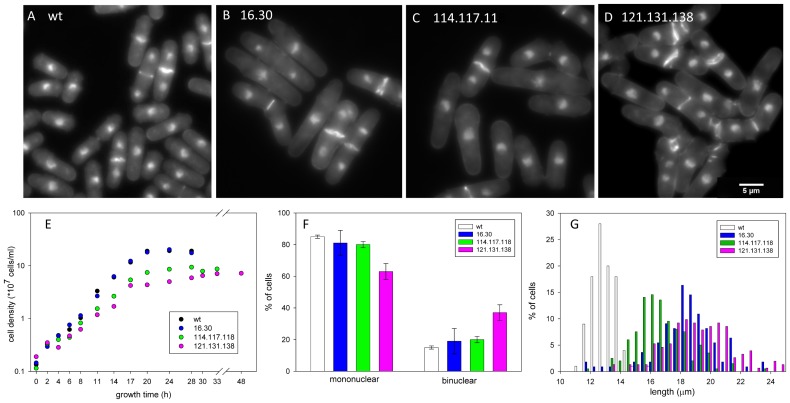
Morphology and nuclear number of gene replacement strains. A–D: representative fields of wildtype and the three mutant strains stained with Calcofluor and DAPI. A. Wildtype cells have typical cylindrical morphology with clearly defined medial septa. B and C. 16.30 and 114. 117. 118 cells are elongated and septa are medially placed but frequently wavy. D. 121.131.138 cells are elongated, often with abnormal shapes and disorganized deposition of septal material. E. Growth curves of wildtype and normal strains. The cell doubling times, calculated from the logarithmic phase of growth, were: wildtype = 2.5 hr, 16.30 = 2.5 hr, 114.117.118 = 2.9 hr, 121.131.138 = 3.3 hr. Strains, 114.117.118 and 121.131.138, reach stationary phase at a lower cell density than wildtype and 16.30. The cell density was calculated using a hemocytometer. The data are averages of two independent experiments. F. Percentage of mononuclear and binuclear cells with standard deviations (two or three independent experiments, n = 400–900 total). All mutant strains show an increase in the fraction of binuclear cells but the increase is statistically significant only for 121.131.138. G. The length distribution of binuclear, septated cells in wildtype and the three mutant strains (two or three independent experiments, n = 100–200 total) indicates that 16.30, 114.117.118 and 121.131.138 cells are longer than haploid wildtype cells because they are diploid. Cells of gene replacement strain 121.131.138 show a broader length distribution than wildtype or either mutant. All strains were grown to mid-log phase in YEA at 30°C.

Our screen of basic parameters of cell growth, size and organization of the actin cytoskeleton shows mutant-specific phenotypes and validates the approach introduced in this work. The wildtype and mutant cells in mid-log growth were analyzed for nuclear number, septum structure and location, overall morphology, and cell length (Figure 5). Wildtype cells show the expected cell shape and size and fraction of binuclear cells (Figure 5A, 5F, 5G) [41]. The septa are well formed and located at the midline. All three mutants exhibit altered shapes and septum morphologies that distinguish them from wildtype and from each other. Both 16.30 and 114.117.118 show a small increase in the fraction of binuclear cells (Figure 5F, not statistically significant), supported by the presence of a minor second peak in the FACS analysis (Figure 4B, 4C). Most septa are well formed and located at the midline, but the septa in both strains are often wavy (Figure 5B, 5C). Together, the results indicate a delay in cell division relative to nuclear replication and separation. The 121.131.138 mutant has a significantly elevated fraction of binuclear cells and abnormal septa, consistent with delayed cell division (Figure 5D, 5F). The presence of a large secondary peak in the FACS (about 50% of the primary peak) supports this conclusion (Figure 4D). In this strain, many septa fail to form discrete structures or form double septa, leading to cells with blebs or branches and nuclear-free cellular fragments after cell division (Figure 5D). Some septa are not perpendicular to the cellular axis. In addition, Calcofluor-stainable material can be found between daughter cells that remain attached and at the new end at the completion of cell division. Similar morphologies were observed when 121.131.138 was overexpressed in the ts background (Figure 3B). These features made scoring of the cells difficult (∼13% of the cells could not be scored for nuclear number), but the cells can eventually divide.

#### Cell size

The 16.30 and 114.117.118 cells have normal overall shapes, whereas 121.131.138 cells are frequently stuck together, making it difficult to distinguish individual cells (Figure 5A–D). The mean lengths of wildtype (12.9±1.0 µ) and the diploid strains (16.30, 18.3 ± 2.0 m; 121.131.138, 19.3 ± 3.0 m; Figure 5G) are similar to those reported for haploid and diploid cells (14.4±0.85 µ and 22.2±1.60 µ respectively [41]. Our lower values may be because our measurements were of ethanol-fixed, stained cells whereas Sveiczer and Mitchison measured living cells growing on agar. The length of 114.117.118 cells (16.8 ± 2.0 m), also a diploid strain, is shorter than expected for a diploid. The results suggest that the *cdc8* mutations affect cell length determination in different ways.

#### Growth rate

**Figure 6:**
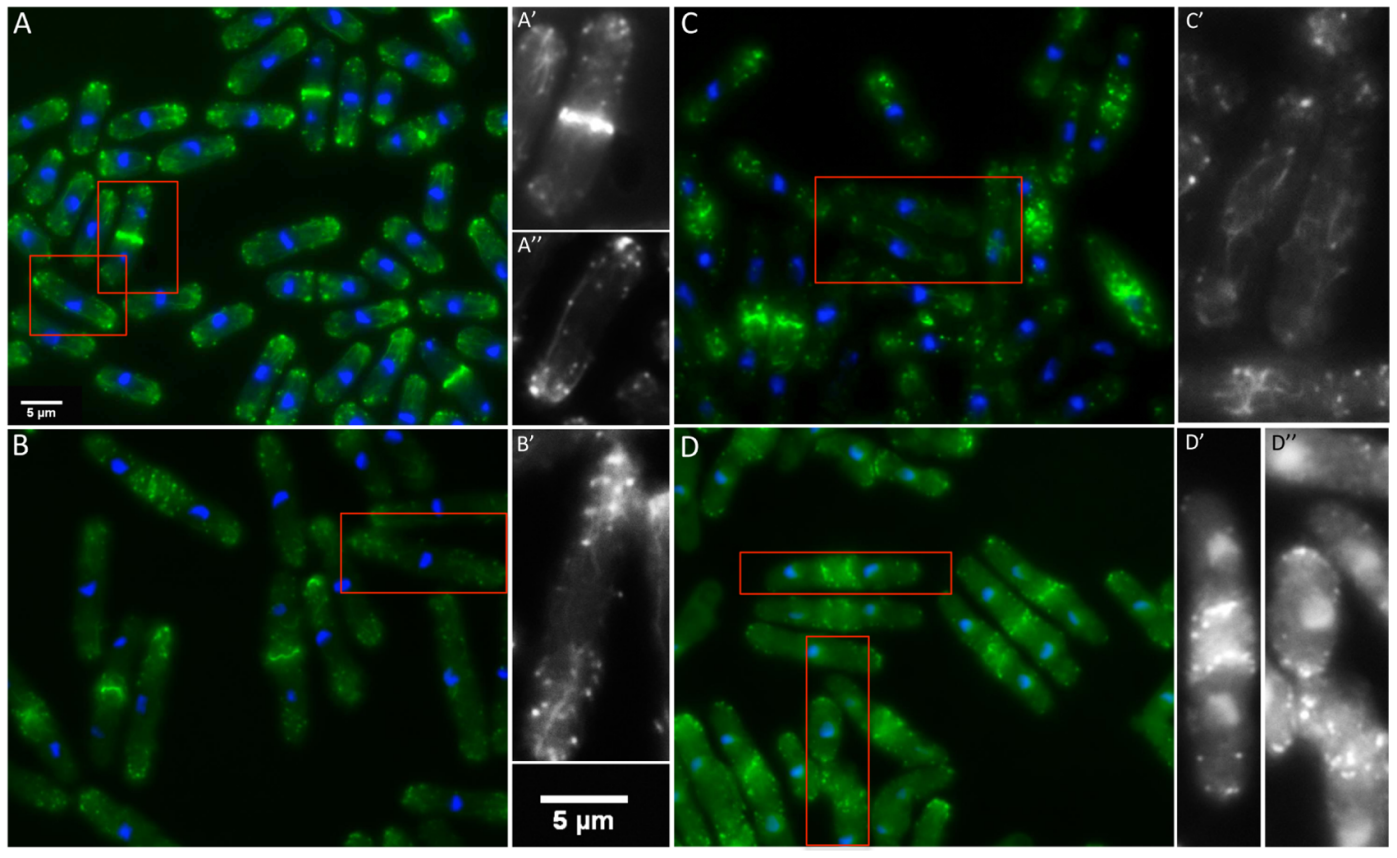
Actin cytoskeleton of gene replacement strains. A–D. The large panels show representative fields stained for filamentous actin (phalloidin, green) and DNA (DAPI, blue). The cells enclosed in red in A–D are enlarged in A’–D’ and A’’–D’’ and show only phalloidin-stained actin. A. Wildtype cells show well-defined contractile rings (A’), long, straight actin cables and mono- or bipolar localization of actin patches (A’’). B. The 16.30 strain exhibits well-formed contractile rings. Cables appeared similar to wildtype in some cells but bent and shortened cable morphologies were frequently observed (B’). C. Strain 114.117.118 showed an abundance of thinner and often reticular cables. Contractile rings often appeared ‘loose’ as seen in the bottom of panel C’. D. In strain 121.131.138 cables are sparse and patches often appear poorly polarized. Distinctive features of 121.131.138 are double contractile rings and rings that appear as an assembly of nodes (D’). Patchy actin aggregations often go along with the shape abnormalities that characterize 121.131.138 (D’’). All strains were grown to mid-log phase in YEA at 30°C.

To learn if the differences in cell length and fraction of cells with two nuclei are related to growth we measured the growth rates of the three mutant strains compared to wildtype (Figure 5E). The cell doubling times were calculated from the slope of the logarithmic phase of growth. Wildtype and 16.30 have doubling times of 2.5 hours. The doubling time for 114.117.118 is 2.9 hours and 121.131.138 is 3.3 hours. Assuming similar doubling times for haploid and diploid cells, the slower growth rate of 121.131.138 is consistent with impaired and delayed cell division as reflected by an increased fraction of binuclear cells. The abnormal position and appearance of septa (Figure 5D, 5F) and the absence of well-formed contractile rings would both contribute to a slower rate of cell division (Figure 6D). By comparison, the longer doubling time of 114.117.118 together with shorter length (Figure 5G) suggests the mutations may affect both size control and timing.

#### Actin cytoskeleton

The actin cytoskeleton is abnormal in all three mutants (Figure 6). In wildtype cells F-actin, visualized using Alexa-phalloidin, is found in actin cables, actin patches and the contractile ring. The actin patches have the expected unipolar or bipolar distribution according to the stage of the cell cycle, discrete contractile rings form at the midline and there are long, straight actin cables, often running most of the cell length.

The *cdc8* mutations affect cable and contractile ring organization in site-specific ways. All three mutant strains show variable polar distribution of actin-containing patches, and the actin cables are wavy and reticular compared to those in wildtype cells (Figure 6B–6D). The effects of the mutations on the actin cytoskeleton are least severe in 16.30 (Figure 6C). Cables are variable in diameter and length, ranging from long and straight (similar to wildtype) to wavy and reticular. The cables in 114.117.118 are thinner and more reticular than in 16.30. In 121.131.138 the cables are not as prominent or consistent as in wildtype or the other two mutant strains. However, the presence of cables and at least partial polar distribution of actin patches indicates that Cdc8p does retain function in all three mutants, in contrast to the *cdc8^ts^* mutants in which actin cables and contractile rings are absent at the restrictive temperature, and polarization of the actin patches is lost (Figure 3B) [36], [37], [38].

The contractile rings are abnormal in all three mutants (Figure 6). The least severe is 16.30, and the most severe is 121.131.138, inferring defects at different stages of contractile ring assembly and function. Nevertheless, the rings are able to constrict. In 121.131.138 the contractile rings predominate as assemblies of what appear to be nodes, similar to the very first stages when myosin assembles into the ring [42]. Often there are two contractile rings, followed by two septa that form on both sides of the midline. Even in cells in which the ring is more discrete, it appears to be an aggregation of nodes, with additional nodes remaining in the cortex near the midline that are not incorporated into the contractile ring. The poorly formed contractile rings in 16.30 and 114.117.118 (Figure 6B, C) have the appearance of rings at early anaphase in wildtype cells when the filaments are not packed and fused, just as the nuclei are beginning to separate, early in spindle pole elongation [36]. However, in both strains, the rings appear to be in the primary stages of formation, even where the nuclei are well separated, as in late anaphase B (spindle pole elongation). Eventually, the rings become more compact without residual cortical nodes, and closure takes place, although in both strains they are less well formed than in wildtype cells. The defect in 114.117.118 is more severe than in 16.30 though double rings and septa, typical for 121.131.138, were never observed.

### Mutations of Conserved *cdc8* Residues Reduce Actin Affinity

**Figure 7:**
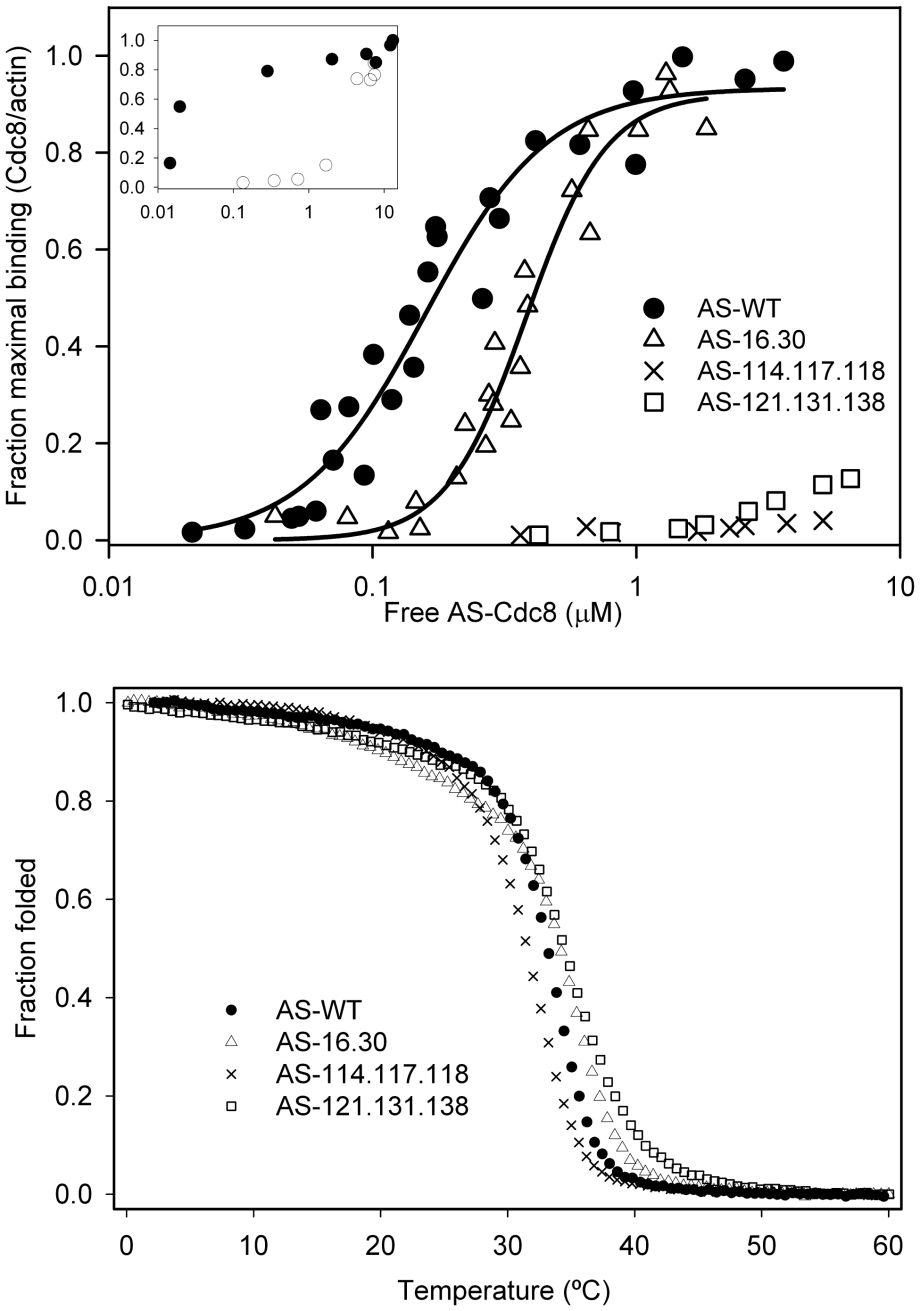
Actin affinity and stability of AS-Cdc8p and mutants. Actin affinity of AS-Cdc8p, wildtype and mutants, measured by cosedimentation as described in Methods (20 mM MOPS, pH 7.0, 150 mM NaCl, 2 mM MgCl_2,_ 5 µM actin). AS-Wildtype, K_app = _6.3×10^6^ M^−1^ (n = 4); AS-16.30, K_app = _2.6×10^6^ M^−1^ (n = 3); the affinity AS-114.117.118 (n = 1) and AS-121.131.138 (n = 1) were too weak to measure. Inset: Actin affinity of AS-Cdc8p (filled circles) and unacetylated Cdc8p (open circles). The affinity of wildtype AS-Cdc8p is ∼500-fold greater than unacetylated Cdc8p. The affinities are too great and too weak, respectively, to calculate the K_app_. Binding conditions: Same as above except 20 mM MOPS, pH 7.0, 100 mM NaCl, 5 mM MgCl_2_ where Tm binds actin with higher affinity. B. Thermal stability determined by measuring the ellipticity at 222 nm between 0–60°C. The ellipticity was normalized to a scale of 0 to 1. The melting temperature (T_M_) is defined as the temperature where the normalized ellipticity is 0.5. The observed T_M_ (n = 1) are: AS-WT = 33.1°C, AS-16.30 = 34.1°C; AS-114.117.118 = 31.5°C, AS-121.131.138 = 34.4°C.

To begin to understand the mechanism for the effect of *cdc8* mutations in living cells, we expressed recombinant Cdc8p in *E. coli* and measured actin affinity and thermal stability. *Cdc8* was modified to introduce AlaSer (AS) at the N terminus, a strategy to increase actin affinity of recombinant Tms that has proven to be effective for striated muscle as well as yeast Tms [43], [44]. Without the N-terminal extension, unacetylated Cdc8p bound poorly to skeletal muscle F-actin, about 500-fold weaker (Figure 7A, inset), in agreement with results from the Maytum laboratory (personal communication) but in contrast to a previous report [25]. The effect of the N-terminal AlaSer on the affinity of Cdc8p for actin is similar to that reported for N-acetylation of skeletal muscle Tm [45].

The actin affinity of recombinant AS-wildtype Cdc8p and its variants was measured by cosedimentation with F-actin. AS-16.30 bound cooperatively to F-actin, with ∼2.4 fold reduced affinity compared to AS-wildtype (Figure 7A; K_app_ of AS-wildtype = 6.3×10^6 ^M^−1^, AS-16.30 = 2.6×10^6^ M^−1^.). Under these conditions, or under conditions where the affinity of AS-wildtype is too tight to measure, the binding of AS-114.117.118 and AS-121.131.138 was too weak to measure at the highest AS-Cdc8p concentrations our assay allows. Even though the intracellular actin concentration is about 10× higher than in our binding assays [46], and operationally even higher because of crowding [47], the reduced actin affinity of AS-114.117.118 and AS-121.131.138 is likely to be a major reason for their cellular dysfunction. The ability of the *cdc8* strains with these mutations to form actin cables indicates residual function that may be supported by other actin binding proteins in the cell. On the other hand, AS-16.30 is likely to saturate actin filaments in the cell and we suggest the observed phenotype results from altered regulatory properties.

### Thermal Stability of Tropomyosins

The thermal stability of wildtype and mutant Cdc8p was determined using circular dichroism to measure the ellipticity at 222 nm between 0–60°C (Figure 7B). There was no major change in stability of the mutants; the small differences from wildtype did not correlate with the effects of the mutations on actin affinity. The main unfolding transition of AS-121.131.138 was slightly less cooperative than wildtype AS-Cdc8p.

## Discussion

In this paper, we present and validate an evolution-based proteomic approach to study structure-function relationships in the conserved, essential cytoskeletal protein, tropomyosin (Cdc8p) in fission yeast. We have identified evolutionarily conserved residues and illustrated their specific importance both in living cells and *in vitro*. The approach will be extended to other conserved surface residues of Cdc8p. The amenability of fission yeast for systems-based and genetic approaches to elucidate function will allow us to combine cellular studies with *in vitro* assays to dissect the specific mechanism of dysfunction in different mutants.

Tropomyosin is involved in numerous cellular processes in fission yeast [23] and more roles for this cytoskeletal regulator may be discovered. For example, Cdc8p is a positive regulator of fission yeast formin (*cdc12*) [6] and of mammalian formins [7]. As with animal myosins, fission yeast myosins can be positively or negatively regulated by Tm. Myo51p, Myo52p, Myo2p, Myp2p are positively regulated by Cdc8p, whereas Myo1p is negatively regulated [48], [49]. Myo2p and Myp2p, together with Cdc8p are required for *in vitro* contraction of the contractile ring [50].The approach taken in the present work provides the framework to define the mechanisms by which Tm carries out these regulatory functions and to understand the consequences in living cells. The advantage of our directed approach based on evolutionary analysis, versus isolation of temperature-sensitive mutants that selects for proteins that are unstable or do not function at high growth temperatures, is that we will identify residues primarily involved in binding rather than folding. The strategy has been successful in identifying animal Tm residues required for binding actin and cooperative regulation of myosin [14], [15], [16], but cellular studies are difficult in a mammalian system.

The three *cdc8* mutants we report here have specific effects on the actin cytoskeleton with reference to actin patch localization, cable and contractile ring morphology, inferring the involvement of the mutated sites in particular cellular functions. All three mutants exhibit delays in contractile ring assembly relative to nuclear division that may relate to transport of materials to the midline and contractile ring, and/or assembly of the ring. The most severe phenotype is seen in 121.131.138 in which the rings appear to be aggregates of nodes rather than filamentous structures. Cytokinesis nodes that are viewed as coalescing into contractile rings contain Mid1p that is important for establishing the midline of the cell in the first stage of contractile ring assembly [51], [52]. The nodes contain numerous other proteins including Cdc12p (formin) and Myo2p and its light chains that are positively regulated by Cdc8p [23], [52], [53], [54], [55], [56], [57], [58], [59]. The delayed contractile ring assembly, patchy appearance, and the residual cortical nodes around the better-formed rings suggest that 121.131.138 is impaired in early stages of ring assembly, and may be a factor in the longer cell doubling time of this mutant. This finding resonates with the SCPR model of contractile ring assembly [54], [60] in which Cdc8p has a role in stabilizing newly formed actin filaments. The 121.131.138 strain shows double contractile rings and septa, and oblique, wavy contractile rings resembling *mid1Δ* and other strains where genes that control positioning of the division plane at the cellular midline are disrupted [61].

The effects of the 16.30 and 114.117.118 mutations on contractile ring appearance are less severe than 121.131.138. The contractile rings are often filamentous in appearance, similar to the aster-like actin structures that Arai and Mabuchi observed at metaphase [36]. These structures may represent actin cables that are nucleated by formin-Cdc12p in other regions of the cell [62] rather than by a SCPR mechanism. Assembly of the non-medial cables requires Cdc12p [6] and compaction into a ring involves Myo2p [56] and Myo51p [63], which are regulated by Cdc8p [6], [12], [48], [58], [59]. An alternate explanation for the contractile ring abnormalities is that actin-Cdc8p filaments containing mutant Cdc8p are more susceptible than wildtype to severing by cofilin-Adf1p [64], a process that is inhibited by Tm [65], [66]. The cell length of the 114.117.118 mutant is shorter than that expected for diploid wildtype cells indicating that the mutation influences size control.

Construction of *cdc8* mutant strains that express fluorescently-labeled myosins, formin, and other cytoskeletal and cell division-related proteins will show whether localization and function of these proteins are affected in the strains expressing mutant Cdc8p. Such analyses, coupled with *in vitro* functional studies would help determine if the *cdc8* mutations affect contractile ring assembly, constriction or both, and suggest residues on Cdc8p that may interact with or regulate the relevant cytoskeletal proteins and lead to the development of mechanistic models.

## Materials and Methods

### Bioinformatics

Phylogenetic trees were constructed from the Tm gene sequences using two different approaches for phylogenetic analyses: maximum-likelihood (GARLI) [27] and Bayesian (MrBayes) [28]. The evolutionary model was JTT+G+F, determined using Prottest [67]. GARLI 1.0 was used for the maximum-likelihood analysis [27]. Branch support was obtained with 1500 bootstrap replicates with 1 search replicate per bootstrap. The 50% majority consensus tree was obtained in PAUP* 4.0 [68]. The Bayesian analysis was done using MrBayes v3.2 [28] with two independent runs of 4 simultaneous chains for 500,000 generations and a sample frequency of 10. The average standard deviation of split frequencies was<0.01 at the end of the runs. The consensus tree was constructed after discarding 25% of the samples. Branch support was obtained as posterior probabilities.

PAML version 4.1 [29] was used to calculate the substitution rates (ω) at individual codon sites using variable site codon models M3 and M7 with 4 rate categories (K = 4) under CODEML with the Bayesian (MrBayes) tree. CODEML was first run under model M0 (no variation in ω among sites) with codon substitution model F61. The branch lengths obtained under model M0 were used for the analysis with variable site models M3 and M7 in order to reduce computational times. The ω values were obtained as the posterior mean, where ω = ω0*PP0+ω1*PP1+ω2*PP2 and so on for each ω category, where PP0, PP1 etc. are the posterior probabilities of categories ω0, ω1 etc, respectively. The average ω value for each site was calculated from the posterior means of the ω values obtained using the two variable site codon models from the Bayesian tree (Table S2 in File S1). In all cases ω<1, meaning there is no neutral or positive selection for any codon.

### Fission Yeast Strains, Plasmid and Genetic Methods

#### Yeast growth and general methods

Strains were grown in EMM (Edinburgh minimal medium, Sunrise Sciences, San Diego, CA) with the appropriate selective supplements (0.0225% adenine, lysine, histidine, uracil) or YEA medium (0.5% yeast extract, 3% dextrose, 0.015% adenine) following standard growth, genetic and cell biology protocols in [69], [70] and other online resources. The strains used and created in this study are listed in Table S5 in File S1. The standard growth temperature was 30°C, 35°C was the restrictive temperature for temperature-sensitive strains, and 25°C was used for permissive growth of temperature-sensitive strains and for other selected procedures. Cells were counted using a hemocytometer. Cellular transformation was carried out using the lithium acetate method [71] and purified plasmid or PCR-generated fragments. Genomic DNA was purified according to [72] with modifications. The breaking buffer was 2% Triton X-100, 1% SDS, 100 mM NaCl, 10 mM TrisHCl, 8.0, 1 mM EDTA; we included an additional chloroform extraction, and RNase A digestion (10 µg/ µl, 30 min at 37°C) in the final step. The *cdc8* sequence was verified in all strains and plasmids used in this study using appropriate primers by sequencing (Genescript, Piscataway, NJ or Genewiz, South Plainfield, NJ).

#### Overexpression in *S pombe*

Cdc8p was overexpressed in wildtype (SP6) and temperature-sensitive cells (*cdc8–27*) [35] using pREP41X, under control of the *nmt* promoter (medium strength) [33], [34]. Mutations were introduced into pREP41X-*cdc8* (cloned between XhoI and BamH1 sites, gift of D. Kovar, University of Chicago), by Mutagenex (Hillsborough, NJ). Following transformation, cells were selected on EMM leu^−^ medium, and expression was repressed by the addition of 10 µM thiamine. The protocol for inducing expression is described in the legend to Figure 3.

#### Marker reconstitution mutagenesis

To make gene replacements of the *cdc8^+^* with selected mutations, we used the method developed by Tang et al. [39] (Figure S2 in File S1). First a fusion PCR fragment was amplified from genomic DNA prepared from a wildtype strain (SP6) using four primers (MRM-P1-MRM-P4, Table S4 in File S1), digested with SalI and BglII and cloned into the SalI and BglII sites of p208H5cdU4^+^ to create p208H5c-cdc8fusion. This plasmid was then digested with PvuII and transformed into YS007 (*h- his5-D1 ura4-D18* ) [73] to place the N-terminal region of *his5^+^* and *ura4^+^* next to the 3′ end of *cdc8*. Transformants were selected on minimal medium lacking uracil, shown to be 5-Fluorootic acid sensitive, and backcrossed twice to confirm the linkage of *ura4^+^* and *cdc8^+^*. Insertion of *his5c* next to the 3′ UTR of *cdc8* was confirmed by genomic sequencing. These strains are SH6, SH7 and SH12, SH13, the latter having been backcrossed to *cdc8-27*, a temperature-sensitive strain.

A second PCR fragment was amplified from genomic DNA using MRM-P4 and MRM-P5 to clone into pH5c at a blunt-end site (PvuII) and BglII sites in pH5c to create pH5c-*cdc8^+^* and to generate a *cdc8^+^his5^+^* coding DNA that is then the template for introducing mutations into *cdc8* (Mutagenex, Hillsborough, NJ). PCR fragments were amplified using P0 and a *cdc8* primer that anneals 5′ to P5, and transfected into SH6, SH7, SH12 or SH13. Transformants were selected on minimal medium lacking histidine and uracil, and backcrossed twice to confirm the linkage of *his^+^* and *cdc8*. The genomic sequence was confirmed. The strains created are SH29, SH30 (wildtype *cdc8^+^*), SH22 (*cdc8^D16A.K30A^*), SH24 (*cdc8^V114S.E117A.H118A^*), and SH25 (*cdc8^R121A.D131A.E138A^*).

The p208H5c-cdc8fusion and pH5c-*cdc8^+^* plasmids were constructed by ExonBiosystems (San Diego, CA).

#### FACS analysis

The ploidy of confirmed strains was analyzed using FACS (M. O’Connell, Mt. Sinai School of Medicine). Cells were grown to mid-log phase and fixed in 70% ethanol. The cells were rehydrated in 50 mM sodium citrate, 100 µg/ml RNAase A and incubated overnight at 36°C. Cells were sonicated for 30 seconds (Bioruptor, Diagenode, Inc., Denville, NJ). Propidium iodide was added to 10 µg/ml. The sample was analyzed on a Becton Dickinson FACScan with Cell Quest Pro software.

### Microscopy

#### Staining

For microscopy cells were fixed at mid-log phase. For staining with DAPI and Calcofluor cells were fixed and stored in 70% ethanol [70], [74]. To visualize the septum, ethanol-fixed cells were washed in PBS and resuspended in 5 µl 50–100 µg/ml Calcofluor (Sigma Life Science, St. Louis, MO) in 50 mM sodium citrate, 100 mM sodium phosphate, pH 6.0) and incubated at ambient temperature for 5 minutes in the dark. For counterstaining with DAPI to visualize the nuclei, the cells were washed and resuspended in 2–5 µl PBS with the addition of 0.5 µl 50 µg/ml DAPI (Sigma Life Science, St. Louis, MO). The samples were incubated for 5 min in the dark at ambient temperature. One µl of stained cells were mixed on a slide with 0.5 µl 1 mg/ml phenylenediamine in 50% glycerol, covered with a poly-l-lysine coated coverslip and sealed with clear nail polish.

Filamentous actin was visualized using Alexa Fluor 488 phalloidin (Life Technologies, Grand Island, NY) [38]. Mid-log phase cells were fixed in 3.7% fresh paraformaldehyde (Electron Microscopy Sciences, Hatfield, PA) for 5 min. at the growth temperature, washed three times in PBS, and stored in PBS with 0.01% NaN_3_ at 4°C for 1 week or less. To stain with phalloidin, 2 µl of fixed cells were permeabilized by vortexing in 100 µl 1% Triton X-100 in PBS for 1 min, and washed 3× with PBS. Alexa-phalloidin was added to the permeabilized cells (4 µl of 0.2 U/µl Alexa-phallodin) and incubated with gentle agitation for 50 minutes at room temperature. Counterstaining with DAPI and preparation of the slides was as described above.

#### Fluorescence microscopy

Epifluorescence images were captured on a Nikon Optiphot 2 microscope fitted for epifluorescence using an Ushio USH-1020H mercury lamp with a Model HB-10101AF power supply and a DS epifluorescence illuminator with Chroma FITC and DAPI filters for imaging Alexa Fluor 488 phalloidin and Calcofluor/DAPI, respectively. We used a Nikon E-Plan 100×/1.25 Ph4DL oil immersion objective lens and a CoolSNAP *fx* camera (Photometrix, Tuscon, AZ). Exposure was controlled by a Uniblitz Model VMM-D1 shutter driver (Vincent Associates, Rochester, NY). IP Lab 4.0.8 (Scanalytics, Inc.) was used to control the microscope and its external devices. Depending on the stain intensity in the examined field, the exposure times were between 100 and 400 ms. Images of the same field were taken at different focal planes to visualize the full thickness of the cells. Binning was set to 1×1. The images were adjusted for brightness and contrast and merged using Image J 1.43 u [75]. Scale bars for all images were obtained by using an AO micrometer with 2 mm divisions subdivided into units of 10 μ.

#### Image analysis

We quantified the nuclear number and cell length in Calcofluor/DAPI stained micrographs using Image J 1.43 u [75]. The length of the cells was measured in pixels and converted to μ using a conversion factor of 1 pixel = 0.0535 µ obtained using the AO micrometer above. The distributions were plotted by rounding the length of cells to the nearest ten pixels and sorting them into groups.

### Expression, Purification and Analysis of Recombinant Cdc8p

Wildtype Cdc8p was expressed in *E. coli* BL21(DE3) using pJC20-*cdc8* (gift of D. Mulvihill, University of Kent) to make unacetylated Cdc8p or pET3a-AS-*cdc8* (gift of M. Lord, University of Vermont) to produce Ala-Ser-Cdc8p. Mutant *cdc8* variants were subcloned from pREP41X-*cdc8* into pET11d [76]. Primer pREP-P1 (Table S4 in File S1) introduces Met-Ala-Ser at the 5′-end together with a NcoI site. The pREP-P2 is homologous to the 3′ end and includes a BamH1 site. These primers were used to create a PCR fragment that was then restricted and ligated into pET11d at the NcoI and BamH1 sites. The Met is cleaved after expression in *E. coli* since the second residue is Ala. Actin was purified from chicken pectoral muscle acetone powder using established methods [77].

Recombinant wildtype and mutant AS-Cdc8p were expressed in *E.coli* BL21(DE3) using the autoinduction method [78] and purified using ammonium sulfate fractionation and anion ion exchange chromatography on DE52 cellulose (Whatman) following established protocols [79], [80]. The method was modified to maximize ammonium sulfate precipation of AS-Cdc8p (45–75% or 30–75%, depending on the form). The purity was evaluated on SDS-PAGE gels and the concentration was determined using the tyrosine difference method [81]. We confirmed the identity of the proteins using electrospray mass spectrometry (W.M. Keck Foundation Biotechnology Resource Laboratory, Yale, New Haven, CT). The measured mass corresponded to the expected mass for the four AS-Cdc8p variants used in this study (AS-Cdc8p-wildtype, measured: 19121, expected: 19122; AS-Cdc8p-16.30, measured: 19020, expected: 19021; AS-Cdc8p-114.117.118, measured: 18985, expected: 18985; AS-Cdc8p-121.131.138, measured 18934, expected 18935).

#### Actin binding assays

Actin 5 µM was mixed with 0.1 µM to 6 µM AS-Cdc8p in 20 mM MOPS, pH 7.0, 150 mM NaCl and 2 mM MgCl_2_ and cosedimented at 20°C at 60,000 rpm in a TLA100 rotor, Beckman TL-100 ultracentrifuge, for 30 min. [79]. The pellets and supernatants were analyzed on 15% SDS-PAGE gels, stained with Coomassie blue, scanned and analyzed using an Image Scanner III (GE healthcare Lifesciences, Piscataway) with Labscan 6.0 and Image Quant TL 7.0 image analysis software. The observed AS-Cdc8p/actin ratio was normalized to 1 by dividing the AS-Cdc8p/actin ratio obtained from densitometry by the AS-Cdc8p/actin ratio observed at saturation. The free AS-Cdc8p in the supernatant was calculated from standard curves for wildtype AS-Cdc8p. The binding constant, K_app_ and Hill coefficient (αH) were determined by fitting the data to the Hill equation using SigmaPlot (Jandel Scientific, San Rafael, CA):
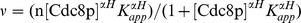
where ν = fraction maximal AS-Cdc8p binding to actin, n = maximal AS-Cdc8p bound, and [Cdc8p] = [AS-Cdc8p]_free_, and αH = Hill coefficient.

#### Circular dichroism measurements

Thermal stability was measured by following the ellipticity of AS-Cdc8p (0.2 mg/ml) at 222 nm in 0.5 M NaCl, 10 mM sodium phosphate pH 7.5, 1 mM EDTA at 0.2°C intervals from 0°C to 60°C using an Aviv model 400 CD-Spectrophotometer at the Robert Wood Johnson Medical School CD facility (Piscataway, NJ). Ellipticity at 222 nm was normalized to a scale from 0 to 1 and a value of 0.5 was defined as the observed melting temperature (T_M_) [82].

## Supporting Information

File S1 **Contains:** Figure S1. Sequence alignment of the fungal tropomyosin genes used in our study. Figure S2. Marker reconstitution mutagenesis strategy. Table S1. List of species and accession numbers. Table S2. ω values of the *S. pombe cdc8* sequence (161 residues). Table S3. Analysis of *cdc8-27* overexpressing wildtype and mutant Cdc8p at 35**°**C. Table S4. List of primers used for MRM and pET vector cloning. Table S5. Strains used in this study. References.
(DOCX)
