# Supplementary material for: A Molecular Evolution Approach to Study the Roles of Tropomyosin in Fission Yeast
Source: PLoS One. 2013 Oct 22;8(10):e76726. doi: 10.1371/journal.pone.0076726 (PMC3805550; doi:10.1371/journal.pone.0076726)
Supplement: File S1 — Contains: Figure S1. Sequence alignment of the fungal tropomyosin genes used in our study. Figure S2. Marker reconstitution mutagenesis strategy. Table S1. List of species and accession numbers. Table S2. ω values of the S. pombe cdc8 sequence (161 residues). Table S3. Analysis of cdc8-27 overexpressing wildtype and mutant Cdc8p at 35°C. Table S4. List of primers used for MRM and pET vector cloning. Table S5. Strains used in this study. References. (DOCX) [file pone.0076726.s001.docx]

**Supplemental Materials**

**Figure S1**

Sequence alignment of the fungal tropomyosin genes used in our study.


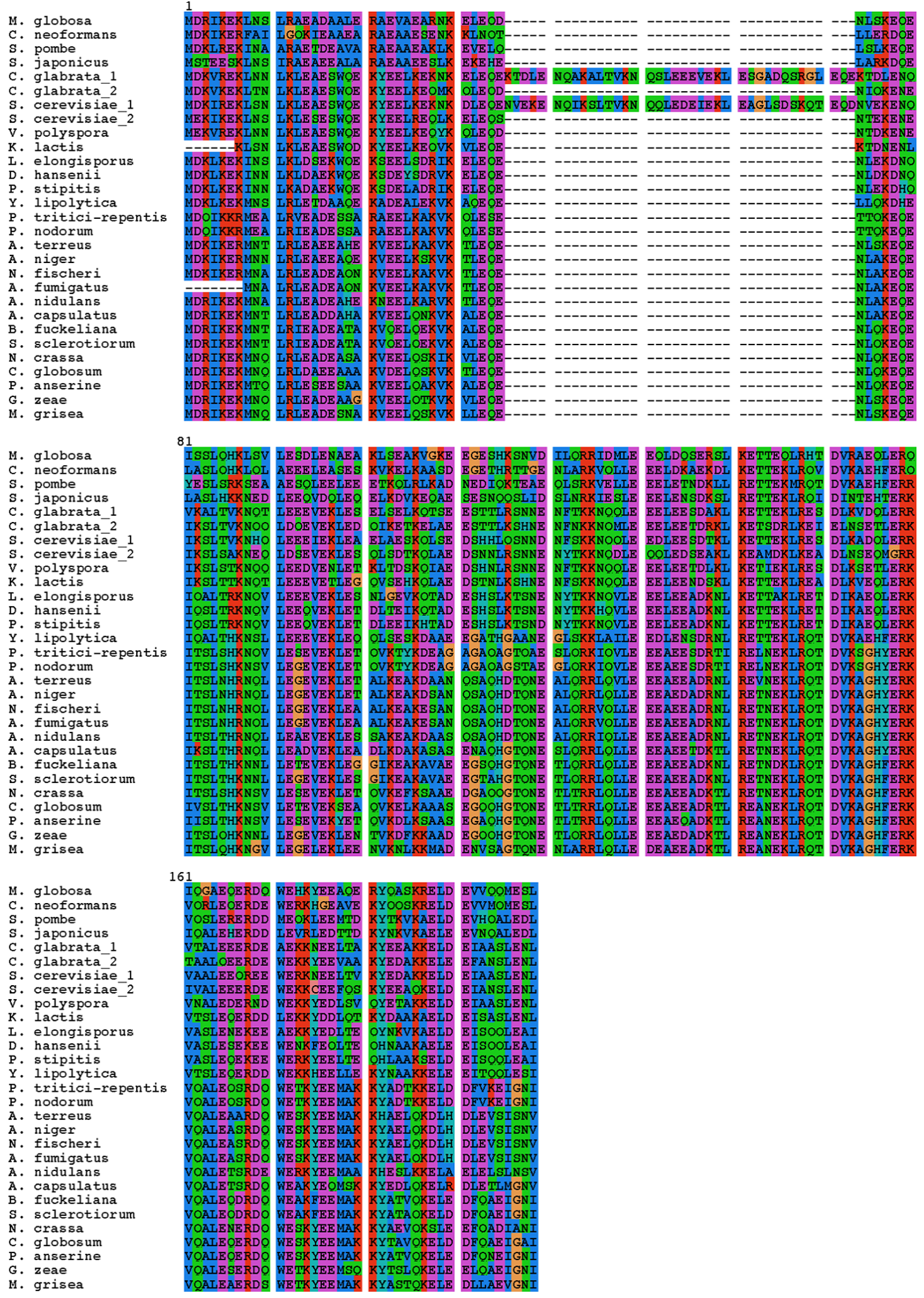


**Figure S2**

Marker reconstitution mutagenesis strategy


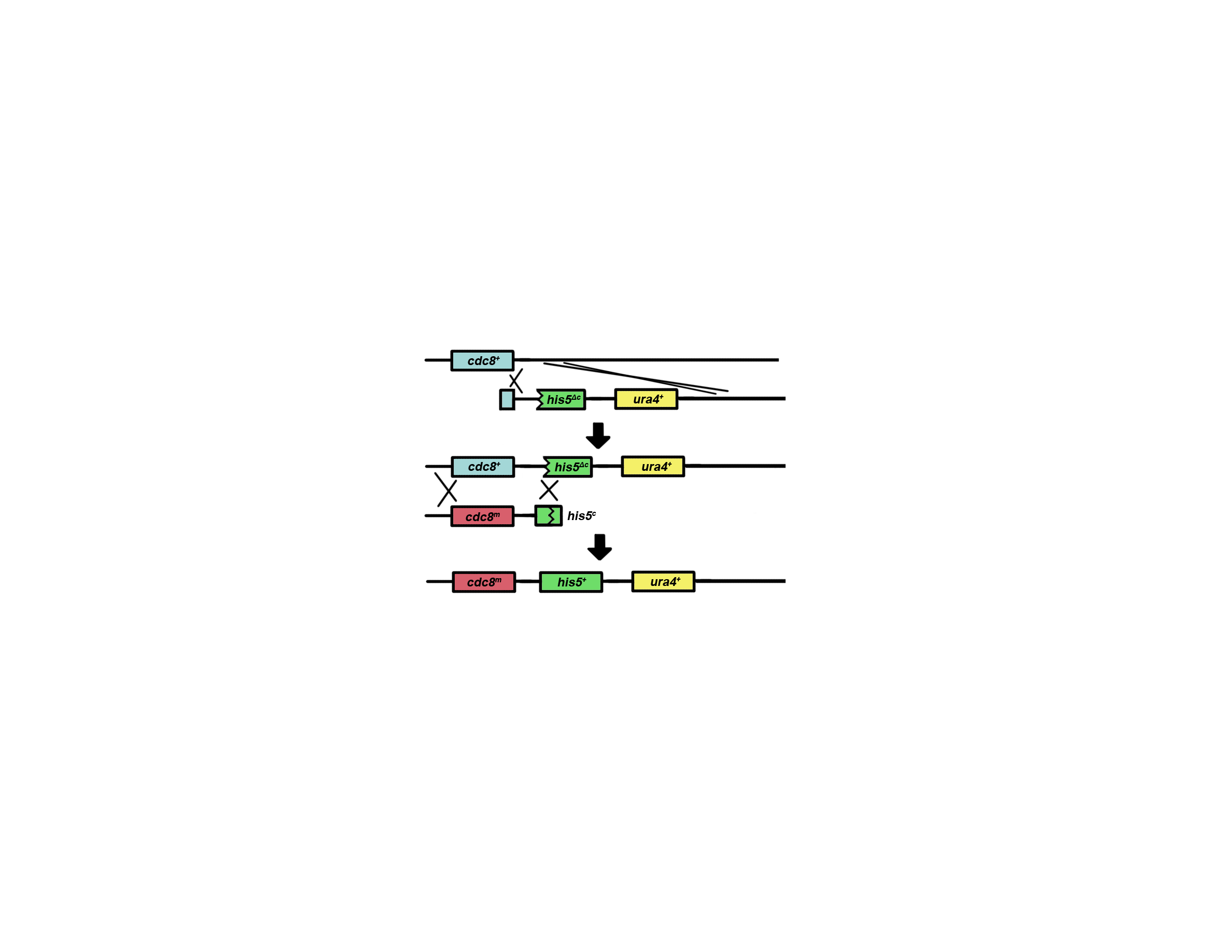


Schematic of the marker reconstitution mutagenesis strategy (Tang *et al.*, 2011). Step 1 illustrates construction of a strain to integrate *his5Δc:ura4^+^* next to the 3’ end of the coding region of *cdc8*. Step 2 illustrates the strategy for introducing *cdc8* mutations and the C-terminal region of the *his5^+^* gene into the strain created in step 1.

**Table S1**

| Species name | Phylum | Accession #s (nucleotide) | # aa |
| --- | --- | --- | --- |
| *Malassezia globosa* | Basidiomycota | XM_001732213 | 161 |
| *Cryptococcus neoformans* | Basidiomycota | XM_769941 | 161 |
| *Schizosaccharomyces pombe* | Ascomycota | NM_001019959 | 161 |
| *Schizosaccharomyces japonicus* | Ascomycota | XM_002175922 | 161 |
| *Candida glabrata (1)* | Ascomycota | XM_447941 | 199 |
| *Candida glabrata (2)* | Ascomycota | XM_449139 | 161 |
| *Saccharomyces cerevisiae (1)* | Ascomycota | NM_001182917 | 199 |
| *Saccharomyces cerevisiae (2)* | Ascomycota | AY558235 | 161 |
| *Vanderwaltozyma polyspora* | Ascomycota | XM_001647278 | 161 |
| *Kluyveromyces lactis* | Ascomycota | XM_454759 | 155 |
| *Lodderomyces elongisporus* | Ascomycota | XM_001525138 | 161 |
| *Debaryomyces hansenii* | Ascomycota | XM_456839.1 | 161 |
| *Pichia stipitis* | Ascomycota | XM_001383892 | 161 |
| *Yarrowia lipolytica* | Ascomycota | XM_505931 | 161 |
| *Pyrenophora tritici-repentis* | Ascomycota | XM_001933125 | 161 |
| *Phaeosphaeria nodorum* | Ascomycota | XM_001793961 | 161 |
| *Aspergillus terreus* | Ascomycota | XM_001213479 | 183 |
| *Aspergillus niger* | Ascomycota | XM_001396179 | 161 |
| *Neosartorya fischeri* | Ascomycota | XM_001261342 | 161 |
| *Aspergillus fumigatus* | Ascomycota | XM_743994 | 154 |
| *Aspergillus nidulans* | Ascomycota | XM_658198 | 161 |
| *Ajellomyces capsulatus* | Ascomycota | XM_001538720 | 161 |
| *Botryotinia fuckeliana* | Ascomycota | XM_001555124 | 161 |
| *Sclerotinia sclerotiorum* | Ascomycota | XM_001596799 | 161 |
| *Neurospora crassa* | Ascomycota | XM_956477 | 161 |
| *Chaetomium globosum* | Ascomycota | XM_001225358 | 161 |
| *Podospora anserina* | Ascomycota | CU640366 (genomic), CAP73204 (protein) | 161 |
| *Gibberella zeae* | Ascomycota | XM_387619 | 161 |
| *Magnaporthe grisea* | Ascomycota | XM_362495 | 161 |

List of species and accession numbers

**Table S2**

ω values of the *S. pombe* *cdc8* sequence (161 residues)

Conserved sites with ω ≤0.02 are shown in bold. The average ω value for each site was calculated from ω values obtained from the trees constructed using two different phylogenetic approaches, Bayesian (MrBayes) (Huelsenbeck and Ronquist, 2001) and maximum-likelihood (GARLI) (Zwickl, 2006) using PAML 4.1 (Yang, 2007).

| Residue  # | Residue  (*S. pombe* sequence) | Heptad  position | ω  (average) |  | Residue  # | Residue  (*S. pombe* sequence) | Heptad  position | ω  (average) |
| --- | --- | --- | --- | --- | --- | --- | --- | --- |
| **1** | **M** | **a** | **0.011** |  | 47 | S | e | 0.172 |
| **2** | **D** | **b** | **0.0115** |  | 48 | R | f | 0.057 |
| 3 | K | c | 0.0535 |  | **49** | **K** | **g** | **0.011** |
| 4 | L | d | 0.0595 |  | **50** | **S** | **a** | **0.012** |
| **5** | **R** | **e** | **0.015** |  | 51 | E | b | 0.174 |
| 6 | E | f | 0.0305 |  | 52 | A | c | 0.132 |
| **7** | **K** | **g** | **0.0125** |  | 53 | A | d | 0.077 |
| 8 | I | a | 0.055 |  | **54** | **E** | **e** | **0.012** |
| 9 | N | b | 0.055 |  | 55 | S | f | 0.174 |
| 10 | A | c | 0.1495 |  | 56 | Q | g | 0.046 |
| 11 | A | d | 0.0545 |  | 57 | L | a | 0.042 |
| **12** | **R** | **e** | **0.017** |  | **58** | **E** | **b** | **0.012** |
| 13 | A | f | 0.1715 |  | 59 | E | c | 0.048 |
| **14** | **E** | **g** | **0.014** |  | 60 | L | d | 0.109 |
| 15 | T | a | 0.075 |  | **61** | **E** | **e** | **0.011** |
| **16** | **D** | **b** | **0.0195** |  | 62 | E | f | 0.174 |
| 17 | E | c | 0.085 |  | 63 | E | g | 0.170 |
| 18 | A | d | 0.073 |  | 64 | T | a | 0.169 |
| 19 | V | e | 0.169 |  | 65 | K | b | 0.165 |
| 20 | A | f | 0.0665 |  | 66 | Q | c | 0.079 |
| **21** | **R** | **g** | **0.0115** |  | 67 | L | d | 0.174 |
| 22 | A | a | 0.0565 |  | **68** | **R** | **e** | **0.011** |
| 23 | E | b | 0.034 |  | 69 | L | f | 0.168 |
| **24** | **A** | **c** | **0.014** |  | 70 | K | g | 0.174 |
| 25 | A | d | 0.106 |  | 71 | A | a | 0.054 |
| 26 | E | e | 0.067 |  | 72 | E | b | 0.068 |
| 27 | A | f | 0.173 |  | 73 | N | c | 0.060 |
| 28 | K | g | 0.057 |  | 74 | E | d | 0.068 |
| 29 | L | a | 0.113 |  | 75 | D | e | 0.174 |
| **30** | **K** | **b** | **0.011** |  | 76 | I | f | 0.157 |
| 31 | E | c | 0.1735 |  | 77 | Q | g | 0.056 |
| 32 | V | d | 0.0635 |  | 78 | K | a | 0.132 |
| **33** | **E** | **e** | **0.0115** |  | 79 | T | b | 0.060 |
| 34 | L | f | 0.039 |  | 80 | E | c | 0.106 |
| 35 | Q | g | 0.0575 |  | 81 | A | a | 0.056 |
| 36 | L | a | 0.0565 |  | 82 | E | b | 0.020 |
| 37 | S | b | 0.1705 |  | 83 | Q | c | 0.074 |
| 38 | L | c | 0.1735 |  | 84 | L | d | 0.048 |
| **39** | **K** | **d** | **0.013** |  | 85 | S | e | 0.172 |
| **40** | **E** | **e** | **0.012** |  | **86** | **R** | **f** | **0.012** |
| **41** | **Q** | **f** | **0.0115** |  | **87** | **K** | **g** | **0.012** |
| **42** | **E** | **g** | **0.015** |  | 88 | V | a | 0.058 |
| 43 | Y | a | 0.052 |  | 89 | E | b | 0.035 |
| 44 | E | b | 0.082 |  | 90 | L | c | 0.165 |
| 45 | S | c | 0.073 |  | **91** | **L** | **d** | **0.014** |
| **46** | **L** | **d** | **0.0145** |  | **92** | **E** | **e** | **0.011** |
| **93** | **E** | **f** | **0.012** |  | 128 | R | f | 0.174 |
| 94 | E | g | 0.026 |  | 129 | E | g | 0.080 |
| 95 | L | a | 0.040 |  | **130** | **R** | **a** | **0.018** |
| **96** | **E** | **b** | **0.115** |  | **131** | **D** | **b** | **0.011** |
| 97 | T | c | 0.070 |  | 132 | D | c | 0.058 |
| 98 | N | d | 0.171 |  | 133 | M | d | 0.128 |
| **99** | **D** | **e** | **0.011** |  | **134** | **E** | **e** | **0.011** |
| 100 | K | f | 0.172 |  | 135 | Q | f | 0.166 |
| 101 | L | g | 0.057 |  | **136** | **K** | **g** | **0.011** |
| 102 | L | a | 0.024 |  | 137 | L | a | 0.069 |
| **103** | **R** | **b** | **0.016** |  | **138** | **E** | **b** | **0.012** |
| **104** | **E** | **c** | **0.011** |  | 139 | E | c | 0.054 |
| 105 | T | d | 0.062 |  | 140 | M | d | 0.056 |
| 106 | T | e | 0.038 |  | 141 | T | e | 0.174 |
| **107** | **E** | **f** | **0.015** |  | 142 | D | f | 0.108 |
| **108** | **K** | **g** | **0.011** |  | **143** | **K** | **g** | **0.012** |
| 109 | M | a | 0.024 |  | 144 | Y | a | 0.036 |
| 110 | R | b | 0.022 |  | 145 | T | b | 0.173 |
| **111** | **Q** | **c** | **0.012** |  | 146 | K | c | 0.174 |
| 112 | T | d | 0.072 |  | 147 | V | d | 0.138 |
| **113** | **D** | **e** | **0.011** |  | **148** | **K** | **e** | **0.012** |
| 114 | V | f | 0.026 |  | 149 | A | f | 0.058 |
| **115** | **K** | **g** | **0.012** |  | 150 | E | g | 0.028 |
| 116 | A | a | 0.060 |  | **151** | **L** | **a** | **0.016** |
| **117** | **E** | **b** | **0.013** |  | 152 | D | b | 0.057 |
| 118 | H | c | 0.044 |  | **153** | **E** | **c** | **0.015** |
| 119 | F | d | 0.060 |  | 154 | V | d | 0.054 |
| **120** | **E** | **e** | **0.012** |  | 155 | H | e | 0.170 |
| **121** | **R** | **f** | **0.014** |  | 156 | Q | f | 0.172 |
| **122** | **R** | **g** | **0.020** |  | 157 | A | g | 0.158 |
| 123 | V | a | 0.036 |  | 158 | L | a | 0.066 |
| 124 | Q | b | 0.056 |  | 159 | E | b | 0.042 |
| 125 | S | c | 0.108 |  | 160 | D | c | 0.056 |
| 126 | L | d | 0.044 |  | 161 | L | d | 0.048 |
| **127** | **E** | **e** | **0.012** |  |  |  |  |  |

| Cdc8p (Tm) overexpressed^1^ | dividing cells^2^  (%) | normally dividing^3^ (%) | cells with septa (%) | normal septa^4^ (%) |
| --- | --- | --- | --- | --- |
| Empty vector | 99.5 ± 0.7 (2) | 5.5 ± 0.7 | 100. (1) | 0. |
| Wildtype Cdc8p | 12.0 ± 4.9 (13) | 95.2 ± 6.4 | 15.9 ± 6.5 (11) | 83.7 ± 12.3 |
| E6A | 9.4 ± 1.0 (3) | 98.1 ± 0.9 | 7.9 ± 1.2 (3) | **54.0** ± 8.0 |
| D16A | 13.1 ± 6.3 (4) | 98.7 ± 2.9 | 12.7 ± 4.7 (3) | **64.5** ± 2.1 |
| E23A | 13.2 ± 4.9 (4) | 99.7 ± 0.6 | 9.1 (1) | 67 |
| D16A.K30A | 16.9 ± 2.4 (4) | 94.0± 2.4 | 17.3 ± 2.6 (4) | 94.4 ± 2.0 |
| K30A | 12.9 ± 4.0 (2) | 90.5 ± 13.4 | 15.0 (1) | 73.0 |
| Q41A | 18.1 ± 9.5 (3) | 99.9 ± 0.2 | 22.0 (1) | 78.0 |
| E58A | 11.0 ± 0. (3) | 100.0 ± 0. | 14.0 (1) | 88.0 |
| E82A^5^ | variable |  | unscorable |  |
| R86A.E93A.R103A.E104A | **27.2** ± 0.8 (4) | 92.1 ± 2.0 | **27.3** ± 0.8 (4) | 92.1 ± 2.0 |
| E104A | 15.0 ± 3.4 (3) | 96.3 ± 3.2 | 17.6 ± 11.9 (2) | 91.6 ± 3.4 |
| E107A.R110A | 14.4 ± 1.3 (2) | 98.5 ± 2.1 | 14.7 (1) | **60.0** |
| V114S.E117A.H118A^5^ | variable |  | unscorable |  |
| R121A | **23.0** ± 6.9 (2) | 93.5 ± 9.2 | **28.2** ± 1.4 (2) | 83.2 ± 3.4 |
| R121A.D131A.E138A | **26.6** ± 0.5 (2) | 91.8 ± 7.3 | **27.4** ± 15.0 (2) | **35.9** ± 4.4 |
| E139A | 15.8 ± 7.7 (2) | 99.4 ± 0.8 | 17.6 (1) | 92 |
| E153A | 15.5 ± 3.8 (2) | 94.4 ± 0.8 | 15.4 (1) | 74 |

**Table S3**

Analysis of *cdc8-27* overexpressing wildtype and mutant Cdc8p at 35 **°**C

^1^ *S. pombe* *cdc8-27* (ts strain) was transfected with pREP41X empty vector or with pREP41X expressing wildtype or mutant Cdc8p. The cells were grown overnight in the absence of thiamine at 25 °C, and then transferred to the restrictive temperature (35 °C) for 18-24 hours before fixation in mid-late log phase of growth. Because of the variable rates of growth and since not all pairs were matched, we did not calculate the statistical difference, but rather used the results to highlight mutations that were obviously different from wildtype (~2 SD) in these parameters and of interest for further study (in bold).

^2^Cells were fixed with DAPI to visualize nuclei. The percent dividing cells is the number of cells with ≥2 nuclei relative to the total number of cells. The means are shown with standard deviation and the number of independent growths counted. Each count was of at least 200 cells.

^3^The percent normal dividing is the number of cells with 2 nuclei relative to the number of cells with ≥2 nuclei.

^4^The percent cells with normal septa (stained with Calcofluor) is the fraction of cells with a single, well-formed and normally positioned septum relative to the total number of cells with septa. Each count was of at least 50 cells.

^5^Overexpression of these mutants resulted in inconsistent growth and variable morphologies from one experiment to the next.

**Table S4** List of primers used for MRM and pET vector cloning

| ***name*** | ***sequence*** |
| --- | --- |
| **MRM-P1** | 5’-***GGCGGCG***TCGACCTCACTTTTTATGGTTTA-3’  SalI |
| **MRM-P2** | 5’-***TTGGCTTCAG***CTGAAGATGAATGTCATTCATACAATAAAGTAAG-3’  PvuII |
| **MRM-P3** | 5’-***TTCATCTT***CAGCTGAAGCCAAGTTGAAAGAAGTTGAACTTC-3’ |
| **MRM-P4** | 5’-GG***C***GG***C***AGA***T***CTTAGCTAGTTGACAAAGTAAGA-3’  BglII |
| **MRM-P5** | 5’-CCCTCCACCATCAAGGAA-3’ |
| **MRM-P0** | 5’-GACGAAGCTCTTTCTAGAAGCGTAGT-3’ |
| **pREP-P1** | 5’-GGCGGCCC***ATGGCATCA***ATG GATAAGCTTAGAGAGAAAATTAAT-3'  NcoI |
| **pREP-P2** | 5'-ACTGGCAAGGGAGACATTCCT-3' |

MRM-P1-MRM-P4 are primers for the marker reconstitution mutagenesis according to Tang et al. (2011). The underlined residues indicate the restriction sites used in the construction of p*208h5c-cdc8fusion* used for construction of SH6 and SH7 (Figure S2, step 1). P5 and P0 are primers to amplify the fragment from p*cdc8H5c* (wildtype or mutant) for transformation of SH6 or SH7. The residues in bold introduce restriction sites or stabilize the oligonucleotide.

The vectors were provided by X. Tang and modified for the current application by ExonBioSystems (San Diego, CA).

pREP-P1 and pREP-P2 are oligonucleotides used to introduce MetAlaSer codons and a NcoI restriction site for cloning into pET11d (Studier *et al.*, 1990) for expression of recombinant AlaSer-Cdc8p. The Met is removed after translation in *E. coli*.

The mutations in pREP41X-cdc8, pET-*cdc8* or p*cdc8H5c* were made by Mutagenix (South Plainfield, NJ).

**Table S5**. Strains used in this study

| Strain | Genotype | Source/reference |
| --- | --- | --- |
| SP6 | *h- leu1-32* | Walworth lab |
| TP9 (KV70) | *h- cdc8-27 his7-366 ura4-D18 leu1-32 ade6-M216* | Nurse et al., 1976 |
| YS007 | *h- his5-D1 ura4-D18* | Tang et al., 2006 |
| FC2320 | *h+ his5-D1 ura4-D18* | Chang lab |
| FC2321 | *h- his5-D1 ura4-D18* | Chang lab |
| SP807 | *h+* *his5-D1 ura4-D18* | Walworth Lab |
| FC363 | *h+ cdc8-27* | Chang lab |
| SH1 | *h+ cdc8-27 ura4-D18* | FC363 x FC2321 |
| SH2 | *h- cdc8-27 ura4-D18* | FC363 x FC2321 |
| SH5 | *h- cdc8-27 his5-D1 ura4-D18* | FC363 x FC2321 |
| SH6 | *h- cdc8^+^::his5^Δc^::ura4^+^  his5-D1 ura4-D18* | This study |
| SH7 | *h-* *cdc8^+^::his5^Δc^::ura4^+^  his5-D1 ura4-D18* | This study |
| SH11 | *h+ cdc8-27 his5-D1 ura4-D18* | SH5 x SP807 |
| SH12 | *h+ cdc8^+^::his5^Δc^::ura4^+^  his5-D1 ura4-D18* | SH6 x FC2320 |
| SH13 | *h-* *cdc8^+^::his5^Δc^::ura4^+^  his5-D1 ura4-D18* | SH1 x SH7 |
| SH22 | *h+* *cdc8^D16A.K30A^::his5^+^::ura4^+^  his5-D1 ura4-D18* | Thus study |
| SH24 | *h- cdc8^V114S.E117A.H118A^::his5^+^::ura4^+^  his5-D1 ura4-D18* | This study |
| SH25 | *h+ cdc8^R121A.D131A.E138A^::his5^+^::ura4^+^  his5-D1 ura4-D18* | This study |
| SH29^1^ | gr*cdc8^+^::his5^+^::ura4^+^  his5-D1 ura4-D18* | This study |
| SH30^1^ | gr*cdc8^+^::his5^+^::ura4^+^  his5-D1 ura4-D18* | This study |

^1^SH29 and SH30 are strains have *cdc8^+^* in the same genetic background as the mutant *cdc8* strains SH22, SH23 and SH25

**References**

Huelsenbeck, J.P., and Ronquist, F. (2001). MRBAYES: Bayesian inference of phylogenetic trees. Bioinformatics *17*, 754-755.

Nurse, P., Thuriaux, P., and Nasmyth, K. (1976). Genetic control of the cell division cycle in the fission yeast Schizosaccharomyces pombe. Mol Gen Genet *146*, 167-178.

Studier, F.W., Rosenberg, A.H., Dunn, J.J., and Dubendorff, J.W. (1990). Use of T7 RNA polymerase to direct expression of cloned genes. Methods Enzymol *185*, 60-89.

Tang, X., Huang, J., Padmanabhan, A., Bakka, K., Bao, Y., Tan, B.Y., Cande, W.Z., and Balasubramanian, M.K. (2011). Marker reconstitution mutagenesis: a simple and efficient reverse genetic approach. Yeast *28*, 205-212.

Tang, X., Jin, Y., and Cande, W.Z. (2006). Bqt2p is essential for initiating telomere clustering upon pheromone sensing in fission yeast. J Cell Biol *173*, 845-851.

Yang, Z. (2007). PAML 4: phylogenetic analysis by maximum likelihood. Mol Biol Evol *24*, 1586-1591.

Zwickl, D.J. (2006). Genetic algorithm approaches for the phylogenetic analysis of large biological sequence datasets under the maximum likelihood criterion.
